# Physical–Chemical Exfoliation of *n-*Alkylamine Derivatives of Layered Perovskite-like Oxide H_2_K_0.5_Bi_2.5_Ti_4_O_13_ into Nanosheets

**DOI:** 10.3390/nano11102708

**Published:** 2021-10-14

**Authors:** Iana A. Minich, Oleg I. Silyukov, Sergei A. Kurnosenko, Veronika V. Gak, Vladimir D. Kalganov, Petr D. Kolonitskiy, Irina A. Zvereva

**Affiliations:** 1 Department of Chemical Thermodynamics and Kinetics, Institute of Chemistry, Saint Petersburg State University, 199034 Saint Petersburg, Russia; st048953@student.spbu.ru (I.A.M.); st040572@student.spbu.ru (S.A.K.); st062004@student.spbu.ru (V.V.G.); irina.zvereva@spbu.ru (I.A.Z.); 2 Interdisciplinary Resource Centre for Nanotechnology, Saint Petersburg State University, 199034 Saint Petersburg, Russia; v.kalganov@spbu.ru; 3 Centre for Innovative Technologies of Composite Nanomaterials, Saint Petersburg State University, 199034 Saint Petersburg, Russia; petr.kolonitckii@spbu.ru

**Keywords:** layered oxides, perovskites, bismuth titanates, exfoliation, nanosheets, coating

## Abstract

In the present work, we report the results on exfoliation and coating formation of inorganic–organic hybrids based on the layered perovskite-like bismuth titanate H_2_K_0.5_Bi_2.5_Ti_4_O_13_·H_2_O that could be prepared by a simple ion exchange reaction from a Ruddlesden–Popper phase K_2.5_Bi_2.5_Ti_4_O_13_. The inorganic–organic hybrids were synthesized by intercalation reactions. Exfoliation into nanosheets was performed for the starting hydrated protonated titanate and for the derivatives intercalated by *n-*alkylamines to study the influence of preliminary intercalation on exfoliation efficiency. The selected precursors were exfoliated in aqueous solutions of tetrabutylammonium hydroxide using facile stirring and ultrasonication. The suspensions of nanosheets obtained were characterized using UV–vis spectrophotometry, dynamic light scattering, inductively coupled plasma spectroscopy, and gravimetry. Nanosheets were coated on preliminarily polyethyleneimine-covered Si substrates using a self-assembly procedure and studied using atomic force and scanning electron microscopy.

## 1. Introduction

Two-dimensional materials obtained from bulk materials have become an important research topic since the inception of graphene technologies [1]. One of the most promising directions in this field is the production of nanolayers via topdown approaches such as the exfoliation of bulk layered oxides. In particular, layered compounds with a perovskite structure are of great interest.

Layered perovskite-like oxides consist of negatively charged perovskite-like layers that fold into a two-dimensional layered structure with cations occupying the interlayer space to compensate for the negative charge of the layers [2,3]. In recent years, layered perovskite-like oxides have attracted great attention due to their structural features and associated anisotropic physical effects. Such compounds exhibit various interesting functional properties such as superconductivity [4,5], dielectric, ferroelectric and piezoelectric properties [6,7,8,9,10], photoluminescence [11,12,13], and photocatalytic activity [14,15,16,17]. In addition, as a rule, they demonstrate high thermal and mechanical stability and can be relatively easily and economically obtained via high-temperature solid-phase synthesis methods [18]. It is important to note that these materials allow useful characteristics to be fine-tuned, since the A sites of layered perovskites can be easily modified with metal ions using a conventional solid-state method [11,19,20,21,22,23].

Such compounds can replace interlayer cations of alkali metals by ion exchange with other cations, in particular, cations of other metals, complex cationic units, or protons, which, in the latter case, allows one to obtain so-called protonated forms [24,25,26,27]. One of the significant features of the protonated forms of perovskite-like oxides is their acidic properties, which allow the intercalation of organic bases to be conducted into their interlayer space, forming organic–inorganic hybrids [27,28,29,30]. Some perovskite-like oxides have been successfully exfoliated into two-dimensional perovskite nanosheets using chemical exfoliation in solutions of bulky quaternary ammonium ions [13,31,32,33,34]. Oxide nanosheets (about 1 nm thick) with the perovskite structure have completely different physical properties than their bulk counterparts. They can serve as multipurpose precursors for creating multilayer films, porous composites, and layered nanocomposites [33,35,36,37,38]. The resulting nanolayers already show intriguing properties with potential uses in photoluminescence [39,40], catalysis [16,41,42,43], and energy storage [44,45].

Even though for some layered perovskite-like oxides, the processes of protonation and subsequent splitting into nanolayers have been studied in great detail, the number of known structures amenable to exfoliation remains rather limited. In this regard, the task of finding new similar compounds is important.

Recently Liu et al. [46] synthesized a new layered Ruddlesden−Popper phase K_2.5_Bi_2.5_Ti_4_O_13_ (KBT_4_) with mixed K/Bi co-occupancy on the perovskite A site, showing stoichiometric hydration and being amenable to form protonated compounds H_2_K_0.5_Bi_2.5_Ti_4_O_13_·yH_2_O [47,48] via substitution of K^+^ by H^+^. The H_2_K_0.5_Bi_2.5_Ti_4_O_13_·H_2_O (HKBT_4_·H_2_O) structure can be described as an alternation of perovskite slabs (K_0.5_Bi_2.5_Ti_4_O_13_) with interlayer protons and water molecules. It was shown that this protonated derivative is capable of intercalation of *n-*alkylamines [49] and grafting of *n-*alcohols in the interlayer space [50]. This work reports on exfoliating HKBT_4_·H_2_O and its *n-*alkylamine derivatives in a tetrabutylammonium hydroxide (TBAOH) aqueous solution to obtain stable suspensions of perovskite nanolayers and their coatings.

## 2. Materials and Methods

### 2.1. Sample Preparation and Characterization

#### 2.1.1. Initial Substances

KNO_3_ (Vekton, St Petersburg, Russia, 99.9%), Bi_2_O_3_ (Vekton, St Petersburg, Russia, 99.9%), and TiO_2_ (Vekton, St Petersburg, Russia, 99.9%) were dried at 200, 600, and 1000 °C, respectively. Methylamine (MeNH_2_, 38% solution in water, Chemical line, St Petersburg, Russia,), ethylamine (EtNH_2_, 70% solution in water, Merck, Darmstadt, Germany), *n*-propylamine (PrNH_2_, Sigma-Aldrich, St Louis, MI, USA, 98%), *n*-butylamine (BuNH_2_, 99.9%, Chemical line, St Petersburg, Russia,), *n*-hexylamine (HxNH_2_, Sigma-Aldrich, St Louis, MI, USA, 99.9%), *n*-heptane (ECOS, Moscow, Russia, 99.9%), TBAOH (40 wt. % solution in water, Acros Organics, NJ, USA); polyethyleneimine (PEI, approx. M.N. 60,000, branched, 50 wt. % solution in water, Acros Organics, NJ, USA) were used as received. 

##### Preparation of Inorganic Hosts 

The starting inorganic matrix, KBT_4_, and its protonated form were prepared by previously reported methods [46,48]. KBT_4_ was synthesized using a solid-state reaction from Bi_2_O_3_, TiO_2_, and KNO_3_. The oxides were taken in stoichiometric ratios; the 20% excess of KNO_3_ was used to compensate for its loss during the calcination due to the volatilization. HKBT_4_·H_2_O was obtained via the ion exchange reaction, which was carried out for 1 week in 1M HNO_3_ at 20 °C.

##### Preparation of Inorganic-Organic Hybrids

Inorganic–organic hybrids with *n-*amines HKBT_4_ × RNH_2_ were obtained by acid–base intercalation in solutions of corresponding *n-*amines. The reactions were performed in optimized conditions reported earlier. [49] Namely, hybrids with methylamine, ethylamine, *n-*propylamine, and *n-*butylamine were obtained via the 24 h reaction at 60 °C using 38% (for methylamine) and 50% aqueous solutions. The reaction with *n-*hexylamine was performed at 80 °C in 50% solution in *n*-heptane. After the reactions, solid products were washed with acetone or *n-*hexane (for HxNH_2_ derivative).

#### 2.1.2. Precursors Characterization

The fact of formation and phase purity of the precursors prepared were controlled using XRD analysis. The detailed qualitative and quantitative characterization of hydrated and dehydrated protonated forms obtained and *n-*alkylamine intercalated hybrids were performed via XRD, Raman, FTIR, and ^13^C NMR spectroscopy, and TG, STA, and CNH analysis, details of which we presented earlier [50]. Starting from the ethylamine derivative, the intercalation of *n-*alkylamines led to a linear increase in the interlayer distance, *d*, compared to that of the hydrated protonated sample, which confirms the fact of the intercalation and the resulting weakening of direct binding of perovskite layers to each other.

#### 2.1.3. Exfoliation

The protonated and amine-intercalated forms obtained were exfoliated using a physical–chemical method that implies the ultrasound treatment of the precursors in an aqueous solution of TBAOH. Specifically, the solid samples were taken in amounts according to a TBA^+^:H^+^ ratio of 1:1 and suspended in 30 mL of 0.004M TBAOH. The suspensions were sonicated using an ultrasonic homogenizer (UP200St) equipped with a 7 mm sonotrode (Hielscher, Teltow, Germany) at a half ultrasound amplitude, A = 50%, for 5 min (total energy ~8000 Ws) twice with intermediate stirring of the suspension at room temperature (~20 °C) for 24 h and 1 week. After the second sonication step, the fraction of large particles containing an unexfoliated residue was separated using centrifugation at 1000 RCF for 60 min.

#### 2.1.4. Concentration Determination

The concentration of suspension was studied firstly by two direct approaches—inductively coupled plasma spectrometry (ICP) analysis and the gravimetry method for the suspension obtained from the *n-*butylamine precursor (for higher concentrations, A = 100% sonication was used). It should be noted that the concentration of obtained suspensions for other layered compounds presented in the literature is often disregarded [31,34,51], and, in several works, it is determined by one of these direct methods [13,39,52]. However, the concentration of the suspensions is of high importance for their future applications and specifically for composite preparations and nanosheets’ deposition.

Gravimetry was performed as follows: the liquid phase was evaporated at 300 °C in a platinum crucible, preliminarily calcined at 900 °C to constant mass. Then, the sediment was calcined at 800 °C for 10 min several times until constant mass was reached. The temperature was chosen according to the complete decomposition of the protonated form into K_0.5_Bi_2.5_Ti_4_O_13_. For ICP analysis, the suspension was mineralized under microwave-assisted heating in a concentrated HNO_3_/HF mixture. It was found that, in general, the gravimetrical method shows slightly higher concentrations compared to ICP analysis. Higher values calculated for K^+^ content compared to Bi^3+^ and Ti^4+^ may be explained by possible incomplete mineralization of the sample. Thus, for further express determination of the concentration, we prepared spectrophotometric calibrations using the nanosheets’ concentration determined by the gravimetrical method.

**Figure A1:**
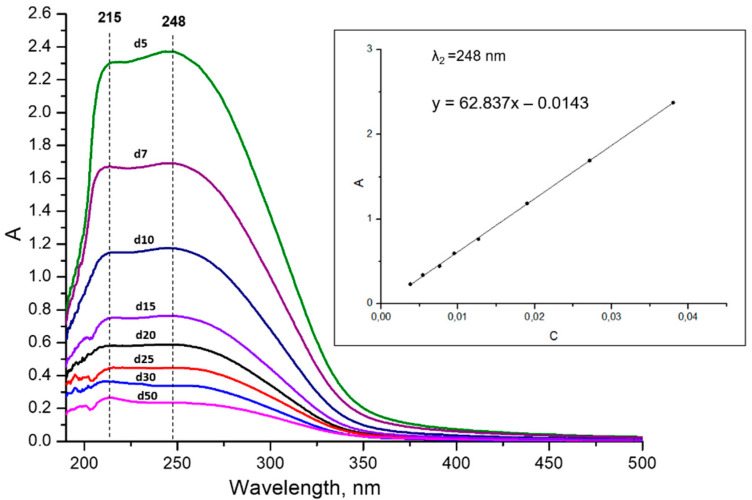
UV–vis spectra of HKBT_4_ × BuNH_2_ suspensions in TBAOH with varied dilution and calibration obtained (inset). The initial concentration of undiluted suspension was 0.195 g/L.

To control and compare the efficiency of exfoliation for different precursors, spectrophotometry calibrations based on those obtained by direct methods concentrations were prepared. For this, the suspension was diluted 5, 10, 15, 20, 25, 35, and 50 times using a 0.004 M TBAOH solution. The UV–vis spectra and calibrations prepared are shown in Figure A1. As may be seen, the shape of the bands upon dilutions is preserved, and two clear shoulders with maximums at 215 and 248 nm are presented. Two spectral maximums were compared, and the maximum at 248 nm was used for the calibration equation. 

**Figure A2:**
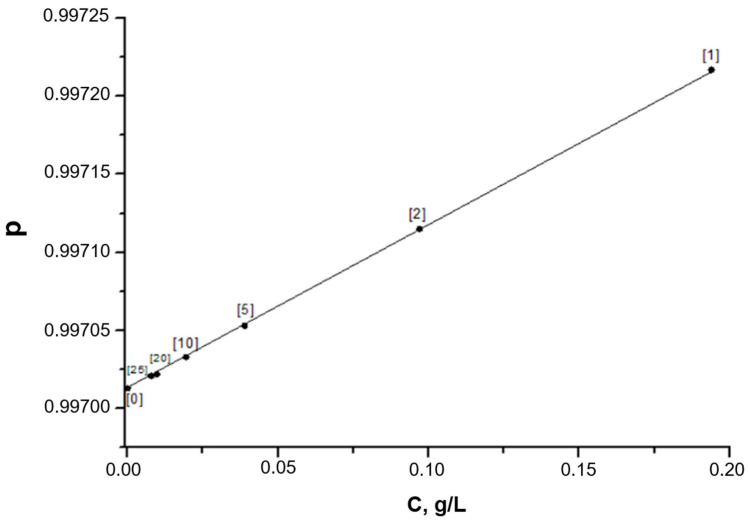
Density calibration obtained for HKBT_4_ × BuNH_2_ suspension diluted to various concentrations (dilution degree presented in [] commas).

Density calibration was also considered as an alternative method that is less dependent on the particles’ size distribution and their morphology. It was found that, in this case, the dependence is also close to linear in the concentration range studied and, therefore, densimetry may be used as a versatile approach to the suspensions’ analysis. The densimetry calibrations obtained are presented in Figure A2. 

#### 2.1.5. Sample Preparation for SEM and AFM Measurements

For further characterization of the nanosheets using SEM and AFM methods, their deposition and flocculation were performed. Nanoparticle reassembly for SEM characterization was carried out following the previously reported method via drop-casting of 1M KCl solution [53]. The precipitate obtained was separated by centrifugation, washed with distilled H_2_O, and dried under CaO.

Nanosheets’ deposition on Si wafers was performed using the self-assembly method shown earlier for other layered niobates and titanates [37,53]. The deposition of the nanosheets was optimized by varying the pH and concentration of the suspensions. The tested concentrations for the original pH (11.9) were 25, 50, 100, 250, 500 and 750 mg/L. Additionally, deposition for suspensions with pH = 9 and concentrations 25, 50, and 100 mg/Lwas performed. Silicon substrates were washed via treatment in 50% HCl/methanol solution at 60 °C for 30 min and the surface was hydroxylated using concentrated H_2_SO_4_ for 30 min at 60 °C. After that, for better adhesion of negatively charged nanosheets, the surface of the substrate was coated with positively charged polymer, PEI, via treatment with 2.5 g/L of its aqueous solution at pH = 9. The coating time was 20 min, according to methods outlined in the literature [37,53]. After coating, the wafers were washed with water and calcined at 250 °C for 30 min to remove surface-adsorbed residual TBAOH. Additionally, the reliability of the coating technique was confirmed with the quartz crystal microbalance–dissipation (QCM-D) experiments. The experiments were carried out on SiO_2_-coated quartz resonators, with a fundamental vibration frequency of 5 MHz. A detector was fixed in the cell with deionized water. The baseline drift was less than 0.1 Hz/h (which corresponds to 1.77 ng/sm^2^). The experiment procedure was as follows: firstly, deionized water was replaced by PEI solution, and the cell was left for 20 min to form the coating; then, the stability of the PEI coating obtained was tested in a flow of water, after which the cell was filled with TBAOH solution. The results of QCM-D experiments showed that PEI coating is already formed after two minutes of treatment. The water flow leads to the partial diffusion of the PEI from the surface; after that, the coating is stabilized. Treatment of the sensor with the TBAOH solution leads to a similar effect caused by the adsorption of TBAOH molecules.

### 2.2. Instrumentation

Powder X-ray diffraction (XRD) analysis of the samples was performed on a Rigaku Miniflex II benchtop diffractometer (Tokyo, Japan) with CuKα radiation, angle range, 2*θ* = 3 − 60°, scanning rate, 10°/min, step, 0.02°. The lattice parameters were calculated on the basis of all the reflections observed using DiffracPlus Topas software (Bruker, Billerica, MA, USA). Concentrations of perovskite nanosheets in colloidal solutions used for building spectrophotometric calibration plots were determined by inductively coupled plasma atomic emission spectroscopy (ICP) on a Shimadzu ICPE-9000 spectrometer (Shimadzu, Kyoto, Japan) after preliminary acid digestion. Spectrophotometric analysis was performed on a Thermo Scientific GENESYS 10S UV–Vis spectrophotometer (Waltham, MA, USA). The pH values of nanosheets suspensions’ media were measured using a laboratory pH-meter Toledo SevenCompact S220 (Mettler Toledo, Greifensee, Switzerland) equipped with an InLab Expert Pro-ISM electrode. Particle size distribution in colloidal suspensions, as well as their ζ-potentials, were determined using the method of dynamic light scattering (DLS) on a Photocor Compact-Z analyzer (Photocor, Moscow, Russia) using DynaLS software (version, Photocor, Moscow, Russia). Morphology of the coated particles was investigated on a Zeiss Merlin (Oberkochen, Germany) scanning electron microscope (SEM). Particle thickness and surface topography were studied using an NT-MDT Integra-Aura (manufacturer, Zelenograd, Russia) atomic-force microscope (AFM) and Gwyddion software (version,, Czech Metrology Institute, Brno, Czech Republic). Quartz crystal microbalance-dissipation experiments were performed on a BiolinScientific Q-Sense E-4 (BiolinScientific, Gothenburg, Sweden) device.

## 3. Results and Discussion

### 3.1. Optimization of Suspensions Preparations

In the present work, physical–chemical exfoliation was used. This approach implies two driving forces for exfoliation: the intercalation of the bulky exfoliation agent into the interlayer space, which facilitates the intercalation of solvent molecules and leads to the swelling of the sample, and subsequent sonication, which generates cavitation bubbles, resulting in high temperature and pressure local zones, which break apart the original samples’ blocks into nanosheets. To determine the impact of these factors on the exfoliation efficiency and to optimize the conditions to prepare highly concentrated suspensions, we considered a row of samples with varied interlayer molecules, namely an as-prepared protonated form, which only includes water molecules in the interlayer space and organically modified samples with preliminarily intercalated *n-*amines: methylamine, ethylamine, *n-*propylamine, *n-*butylamine, and *n-*hexylamine. In addition, the duration of the intermediate stirring between sonication steps was varied for some of the samples.

**Figure 1:**
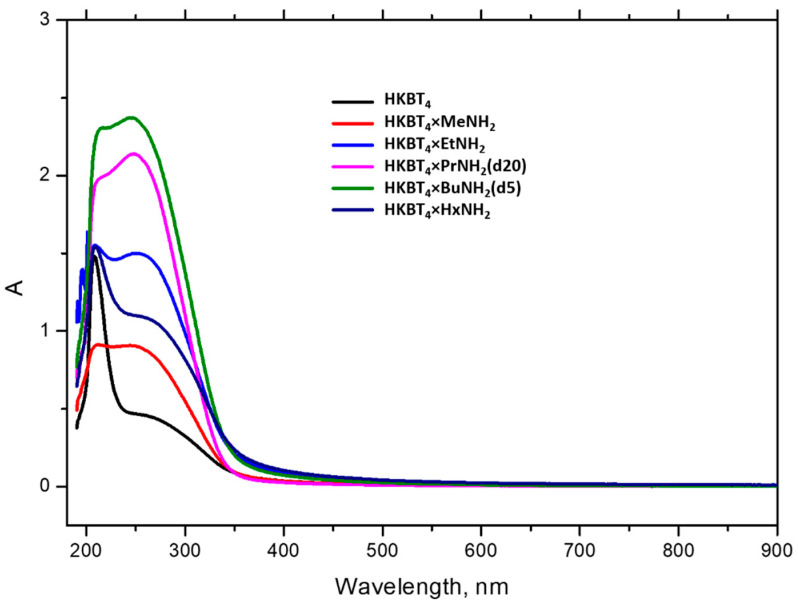
UV–vis spectra of suspensions obtained from protonated titanate HKBT_4_·H_2_O and its inorganic–organic hybrids.

Examples of UV–vis spectra, obtained for the suspensions of protonated and *n-*amine-intercalated samples with 24 h holding, are presented in Figure 1. As it may be seen in the case of organically modified precursors Me-Bu, two wide shoulders with maximums at 215 and 248 nm are presented on spectral bands, which may be the result of the size distribution of particles in the suspension. In the case of the protonated and *n-*hexylamine samples, a sharp peak at 210 nm appears. It should also be noted that visible agglomeration was detected for these suspensions, which was later additionally confirmed by DLS measurements for the dispersed protonated form. So, we associate the appearance of this band with the formation of bigger spherical agglomerates providing different scattering effects. In the case of the *n-*hexylamine sample, the floating and poor mixing of the powder in the TBAOH water solution were observed, which is most possibly caused by the strong hydrophobicity of the surface-adsorbed *n-*hexylamine. Although, as it may be seen from the spectra, sonication still made its partial exfoliation possible, implied by the presence of the wide shoulder, as in the case of other *n-*amine intercalates. The sample obtained was strongly unstable and the residue of floating agglomerates could not be separated by centrifugation, so this sample is not considered further in this work.

The concentrations of the suspensions prepared from the row of amine-intercalated samples with intermediate stirring for 24 h and 1 week are presented in Table 1. In the case of 24 h experiments, exfoliation was performed three times with different precursors, prepared under the same conditions, and the average concentration is presented. As it may be seen, the concentration insignificantly varies for suspensions, prepared using stirring for 24 h and 1 week, so it may be concluded that the insertion of exfoliant and solvents molecules is not time-dependent for these samples. In contrast, the choice of precursor for exfoliation strongly influences the exfoliation efficiency. In all of the cases, the most concentrated suspensions could be prepared using the *n-*propylamine and *n-*butylamine intercalates. The highest nanosheet concentration achieved using the *n-*propylamine derivative was about 780 mg/L. Earlier, we tested other Ruddlesden–Popper titanates, HLnTiO_4_ and H_2_Ln_2_Ti_3_O_10_ (Ln = La, Nd) [54], the exfoliation of which was more efficient for the methylamine derivatives in comparison with *n-*butylamine ones, so we expected similar results for the present bismuth titanate. This was mainly explained by the stronger methylamine affinity to water, compared to less hydrophilic longer-chain *n-*amines, which leads to the more effective intercalation of water molecules during the stirring period, promoting subsequent exfoliation. Another possible reason may be associated with stronger alkyl–alkyl interactions between alkyl chains in the interlayer space for larger *n-*amines, preventing the lamination of the layered structure. The present work uses similar approaches to compare the exfoliation process for two different matrices and, as a result, it may be concluded that the choice of precursor for each matrix should be found experimentally rather than theoretically. 

### 3.2. DLS Measurements

**Figure 2:**
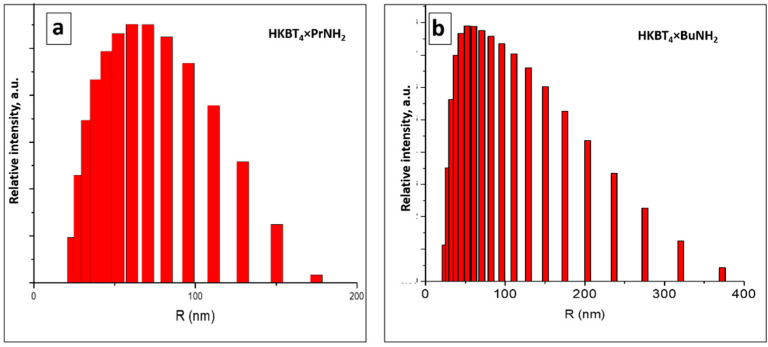
Particles size distribution for selected (**a**) *n*-propylamine and (**b**) *n*-butylamine non-aggregated samples.

Average sizes of particles and size distribution in the suspensions prepared from various precursors, determined by DLS measurements, are shown in Table 2 and Figure 2. DLS measurements were performed for all of the suspensions the next day after preparation, and suspensions were shaken before the measurements. The presented results for samples with the narrowest size distribution (for *n*-propylamine) and wider size distribution (for *n*-butylamine) are refracted in the calculated average size for each sample. As it may be seen, in all of the cases, the average particle sizes were about 100 nm, except for slightly decreased sizes for HKBT_4_ × PrNH_2_ suspension. The poor stability of the suspension prepared from protonated sample led to the aggregation of the particles into ~2 µm agglomerates after 24 h. However, the resonication right before the measurement shows that the original sizes of the particles correlate with the ones for amine-containing samples.

### 3.3. Stability of Nanoparticles Suspensions

**Figure 3:**
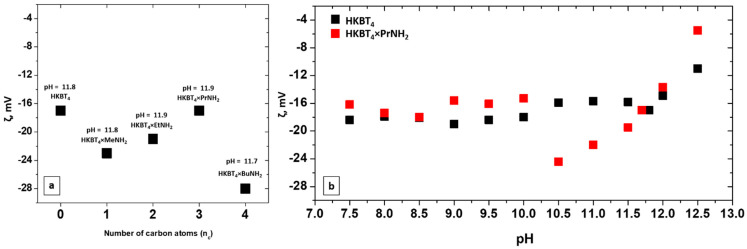
Potentials for (**a**) suspensions, prepared with various inorganic–organic precursors, (**b**) HKBT_4_ (black) and HKBT_4_ × PrNH_2_ (red) suspensions with varied pH.

The stability of the suspensions obtained is an important characteristic defining the possibility of their storage and practical use. Moreover, stability should be considered for the further deposition of particles. In addition to visual and spectrometric control of suspension stability over time and during centrifugation (as we use centrifugation to separate unexfoliated residue, it also serves as a factor of the detected “exfoliation efficiency”), the aggregative stability of suspensions was also evaluated by measuring zeta potentials. ζ-potentials were measured for as-prepared suspensions from various precursors and for suspensions, prepared from protonated and *n-*propylamine intercalated forms at different pHs. ζ-potentials for suspensions prepared from various amine-intercalated precursors are presented in Figure 3a. As it may be seen, the ζ values obtained vary in the range from −17 to −27 mV, which corresponds to the average stability of metal oxide suspensions. However, the concentrated suspensions obtained did not show any visible agglomeration after 2 months of undisturbed storage (in the case of Me-Bu samples). 

The main factors affecting ζ-potential considered are the pH of the suspensions, surface conductivity [55], and the concentration of suspensions [56]. It was shown earlier that strong dilution may lead to the lower stability of suspensions, which was observed in the case of the exfoliated protonated form. It should also be noted that the most concentrated suspension obtained using the *n-*propylamine sample showed a similar value for zeta potential. Nevertheless, practically, this suspension showed high stability.

The dependence of ζ-potentials on pH for the suspensions obtained from protonated and *n-*propylamine forms is presented in Figure 3b. In both cases, the original pH of the suspensions was ~11.8–11.9 and it was lowered by the dropwise addition of HCl and raised using a KOH solution. The step was 0.5 and the maximum studied pH was 12.5 due to the visible strong agglomeration of the particles at this pH. Both of the suspensions show similar values of ζ-potentials at their original pH (~17 mV). The values for the protonated sample at pH = 7.5–11.8 vary in the range of ~16–19 mV and the lowering is observed at higher pH with the minimum by module at pH = 12.5 (ζ = −11 mV). A similar plot is obtained for the *n-*propylamine sample in the pH range of 7.5–10 with the local maximum (by module) at pH = 10.5 (ζ = −24 mV). A more crucial decrease in ζ-potential is observed at a high pH with minimal value ζ = −5.5 mV). 

The pH of the obtained suspensions was also considered to verify that the difference in stability and obtained concentration does not arise from the difference in pH. In all of the cases, the pH of the suspensions prepared with 24 h stirring was in the range of ~11.7–11.9, which corresponds to the original pH of the TBAOH solution (11.8), while the pH for suspensions prepared with 1 w stirring was found to be in the range of ~9.5–9.9, which implies that pH does not depend on the precursor chosen for exfoliation and the concentration of suspensions obtained. Thus, there is no evident correlation between the pH of suspensions and exfoliation efficiency in the pH range studied.

### 3.4. Particles Deposition on Silicon Wafers

**Figure 4:**
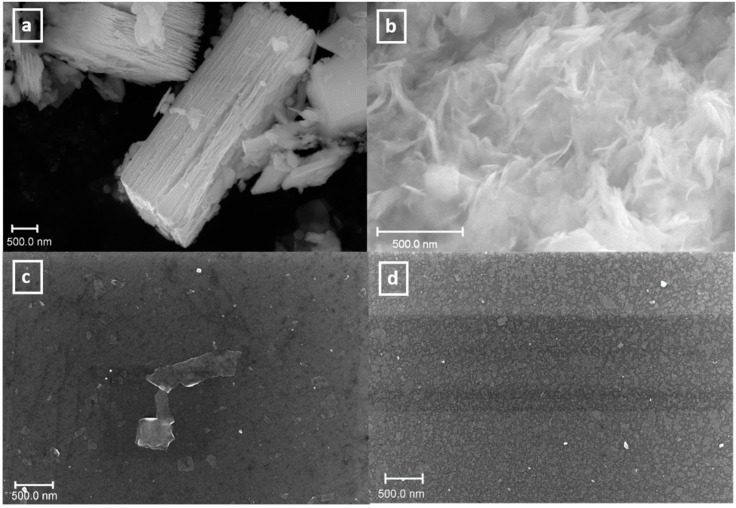
SEM images for initial HKBT_4_ PrNH_2_ sample (**a**); reassembled after exfoliation sample (**b**); Si substrate coating with 50 mg/L suspension at pH = 11.9 (**c**) and Si substrate coating with 750 mg/L suspension at pH = 11.9 (**d**).

Deposition of the samples was performed from suspensions prepared using the *n-*propylamine precursor, as they had the highest original concentration. In the self-assembly approach used before for niobates and titanates [37,53], relatively diluted suspensions were used and the pH was usually adjusted to pH = 9, as it favors the presence of a higher percentage of cationic groups in the PEI coating [57]. In this work, we varied the concentration, and in some cases, the pH of suspensions to optimize particles’ deposition, as such experiments have not been performed before with bismuth titanates. The efficiency of deposition and the lateral size and thickness of the obtained particles were studied using SEM and AFM. SEM images for selected samples are shown in Figure 4.

The bulk powder precursors obtained had a plate-like morphology with relatively big lateral sizes. After exfoliation and reassembly, the agglomerated nanosheets were formed. SEM of the wafers obtained showed that in the case of low concentrations, only some single particles could be detected on the surface and using high concentrations leads to the formation of a more dense coating. 

**Figure 5:**
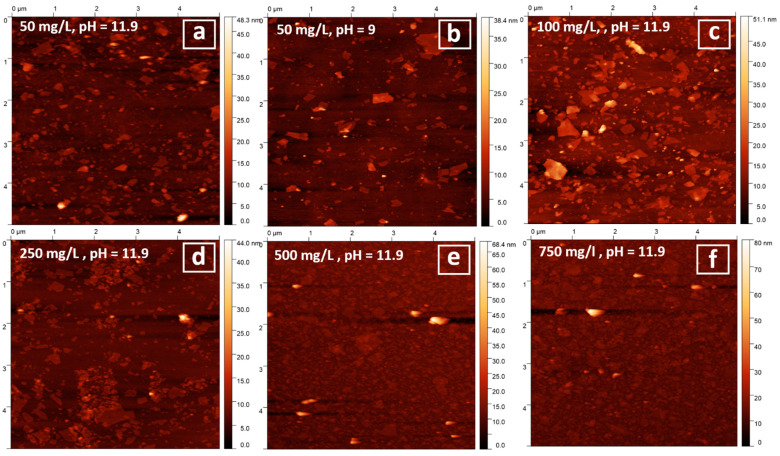
AFM images for coatings obtained in various conditions: 50 mg/L, pH 11.9 (**a**); 50 mg/L, pH 9 (**b**); 100 mg/L, pH 11.9 (**c**); 250 mg/L, pH 11.9 (**d**); 500 mg/L, pH 11.9 (**e**); 750 mg/L, pH 11.9 (**f**).

The successful deposition of particles was further proven using AFM. The corresponding images for selected samples are shown in Figure 5.

**Figure 6:**
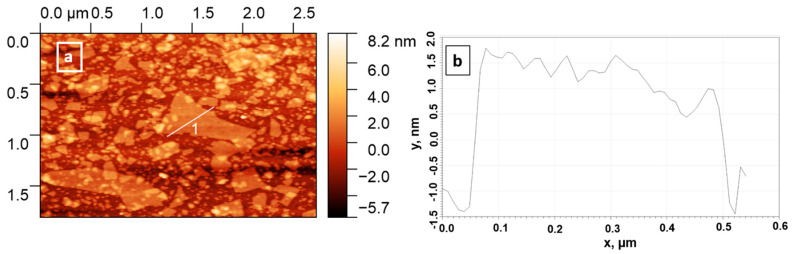
AFM image area (**a**) and height profile for a selected particle (**b**) for 500 mg/L, pH 11.9 suspension.

**Figure A3:**
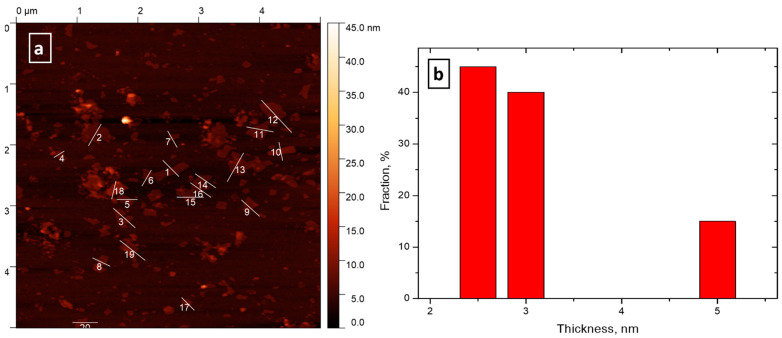
(**a**) AFM image for particles deposed from 250 mg/L suspension with marked particles used for thickness distribution calculations and (**b**) obtained thickness distribution.

The deposition of low concentrated samples (25 mg/L) was ineffective and almost no particles were detected on the wafers in both of the cases (at original pH = 11.9 and pH = 9). An increase in suspensions’ concentrations generally was shown to result in more dense coatings. Single deposited particles may be observed for the samples, prepared using 50, 100, and 250 mg/L suspensions. The thickness of the particles obtained was found to be in the range of 2.5–5 nm, which corresponds to the monolayers and bilayers of the starting inorganic matrix. (Figure 6 and Figure A3). Using the highly concentrated suspensions (500 and 750 mg/L) resulted in the formation of a relatively dense coating of the silicon surface.

## 4. Conclusions

The physical–chemical exfoliation of a new Ruddlesden–Popper bismuth titanate, H_2_K_0.5_Bi_2.5_Ti_4_O_13_, and its inorganic–organic hybrids with *n-*alkylamines was studied for the first time in an aqueous solution of TBAOH. Although it was possible to carry out the process of splitting and obtain a suspension of nanolayers for all samples, it was shown that the choice of precursor has a strong impact on the efficiency of exfoliation. To determine the concentration of the obtained suspensions, the two direct approaches—ICP analysis and the gravimetry method—were used. For express analysis, indirect UV–vis spectroscopy and the densimetry method were proposed. The most concentrated suspensions (up to 770 mg/L) after the removal of the unexfoliated part of oxide via centrifugation were obtained using the *n-*propylamine derivative. At the same time, the initially protonated derivative without intercalated amine was shown to have the worst exfoliation ability and stability of the resulting suspension. No correlation was found between ζ-potentials of the suspensions obtained and their concentrations for various samples, which implies that the stability of the resultant suspension does not influence the exfoliation procedure, considering the possible precipitation of unstable exfoliated particles during the centrifugation. According to the DLS data for the suspension obtained from the n-propylamine derivative, the average particle size lies at around 65 nm, which is in agreement with SEM and AFM data for the deposited particles. The negatively charged nanosheets’ deposition on the surface of the Si substrate coated with positively charged polymer, PEI, was studied, considering the concentration, and in some cases, the pH of the suspension. As a result, the dense coatings were obtained using suspensions with high concentrations for the first time for Ruddlesden–Popper-type titanates. The thickness of the major particles deposited was found to be around 2.5–3 nm, which corresponds to the single-layer thickness of the starting inorganic matrix; this indicates that under the conditions used, it is possible to obtain suspensions of monolayers of the corresponding oxide.

Thus, the results presented in this work expand the range of objects for the creation of oxide nanolayers. The resulting nanolayers and coatings can be used as functional materials for catalysis and electronics.

## Figures and Tables

**Table 1:** Concentrations of suspensions obtained from various inorganic–organic hybrids with 24 h and 1 week intermediate stirring time calculated from UV–vis spectra.

Sample	Concentration (24 h), mg/L	Concentration (1 w), mg/L
HKBT_4_ × MeNH_2_	18	13
HKBT_4_ × EtNH_2_	26	23
HKBT_4_ × PrNH_2_	730	770
HKBT_4_ × BuNH_2_	156	245

**Table 2:** Average sizes of prepared nanoparticles in suspensions determined by DLS.

Sample	Average Size, nm
HKBT_4_ (aggregated)	~2000
HKBT_4_ (resonicated)	96
HKBT_4_ × MeNH_2_	77
HKBT_4_ × EtNH_2_	83
HKBT_4_ × PrNH_2_	65
HKBT_4_ × BuNH_2_	88

## Data Availability

The data presented in this study are available in the article.
